# The effects of management on vegetation trajectories during the early‐stage restoration of previously arable land after hay transfer

**DOI:** 10.1002/ece3.5798

**Published:** 2019-12-06

**Authors:** Myriam Garrouj, Didier Alard, Emmanuel Corcket, Lilian Marchand, Marie‐Lise Benot

**Affiliations:** ^1^ INRA BIOGECO UMR 1202 Univ. Bordeaux Pessac France; ^2^ Present address: LyRE Research and Development Center of SUEZ Talence France

**Keywords:** community‐related metrics, ecological restoration, grazing, mowing, trajectory

## Abstract

The restoration of floodplain grasslands has benefited from many studies of the underlying mechanisms. Among the operational tools that resulted, hay transfer is now used increasingly to alleviate the effects of limited seed dispersal and recruitment. To improve this method, we still need to understand how it can affect restoration trajectories, and particularly their direction and magnitude during the early stages of restoration. Based on concepts from the field of community ecology theory, we investigated the effects of early‐stage management through grazing or mowing on restoration trajectories after soil harrowing and hay transfer. We established a randomized block design experiment and quantified several community‐related metrics to formalize restoration trajectories for 3 years after hay transfer on a previously arable alluvial island in southwestern France. Whatever the management treatment, the species richness and evenness were significantly higher in hay‐inoculated than in control plots. This effect was linked to the recruitment of species originating not only from the reference grassland through hay transfer, but also from the seed bank, a well‐known effect of soil harrowing. Although generally oriented toward the reference grassland, the origin, direction, and magnitude of the trajectory of hay‐inoculated plots all depended on the management applied. Sheep grazing applied at the same time as hay transfer enhanced the recruitment of reference species as from the first experimental year, because it controlled aboveground competition and maintained the window of opportunity open for a sufficiently longer period of time. Our findings show that the type of management applied simultaneously to hay transfer influences the origin of a grassland trajectory, while its direction and magnitude are dependent on the management applied in subsequent years. Grazing immediately after hay transfer may be appropriate to accelerate the recruitment of species from the reference grassland.

## Graphical abstract

The restoration of floodplain grasslands has benefited from many studies of underlying mechanisms. Among the resulting operational tools, hay transfer is increasingly used to alleviate the effects of limited seed dispersal and recruitment. To improve this method, we still need to understand how it can affect restoration trajectories, especially their direction and amplitude during the first stages of restoration. Our findings show that management influences mostly the origin and amplitude rather than the direction of grassland trajectory after hay transfer.

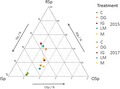

## 1 INTRODUCTION

During recent decades in Europe, agriculture intensification has led to dramatic losses of natural and seminatural habitats (Henle et al., 2008; Tscharntke, Klein, Kruess, Steffan‐Dewenter, & Thies, 2005). In large river floodplains, this process started with the loss of pristine wetland habitats due to agricultural use and the construction of river embankment during past centuries, thus affecting the seminatural grasslands that resulted from traditional agricultural land use. These threatened ecosystems can offer high levels of species diversity and are considered as priority habitats by the European Natura 2000 network (Henle et al., 2008; Verhoeven, 2014, Council Directive 92/43/EEC). In that context, river floodplains have since been targeted for ecological restoration programmes according to two major options with respect to “reference ecosystems”: the pristine organization of riverine systems on the one hand and traditionally used seminatural grasslands on the other (Dufour & Piégay, 2009; Poudevigne, Alard, Leuven, & Nienhuis, 2002; Verhoeven, 2014).

The restoration of grasslands from previously arable lands has been the subject of numerous studies focused on the ecological mechanisms underlying restoration successes or failures (Muller, Dutoit, Alard, & Grévilliot, 1998; Pywell et al., 2002; Török, Vida, Deák, Lengyel, & Tóthmérész, 2011). Experimental approaches have considerably strengthened the conceptual bases of restoration ecology by testing hypotheses from the field of community ecology (Wainwright et al., 2018) and exploring key processes in restoration operations such as biotic interactions or disturbances (Buisson, Corcket, & Dutoit, 2015). One of the principal processes that affects restoration success is the limited dispersal and recruitment of plant seeds in a context of habitat fragmentation (Pywell et al., 2002; Woodcock, McDonald, & Pywell, 2011), particularly after a long history of intensive agricultural practices that has reduced the number of floodplain grassland species seeds stored in the seed bank (Bischoff, Warthemann, & Klotz, 2009; Hedberg & Kotowski, 2010; Scotton, 2016).

This has led to several restoration methods (Kiehl, 2010; Török, Vida, et al., 2011), among which hay transfer is being increasingly applied and has the potential to be used worldwide (Albert et al., 2019; Coiffait‐Gombault, Buisson, & Dutoit, 2011; Hedberg & Kotowski, 2010; Klimkowska, Diggelen, Bakker, & Grootjans, 2007). Although the hay transfer method is now well established for the restoration of seminatural grasslands, there remain gaps in our knowledge and unexplored issues, especially regarding the early stages of the restoration process and their effects on long‐term dynamics. Many studies have presented short‐term community trajectories after restoration; the advantage is that they have described contrasted and dramatic changes but they have been limited by weak long‐term predictability from these short‐term results (Auestad, Austad, & Rydgren, 2015). The question of whether initial seed transfer can only accelerate a predicted succession or has a major and permanent influence on the long‐term trajectory is a crucial and still underestimated issue (Young, Petersen, & Clary, 2005). In particular, we still need to understand whether and how initial restoration and management choices (e.g., grazing and mowing) can explain different trajectories (Pywell, Meek, Webb, Putwain, & Bullock, 2011; Rinella, Espeland, & Moffatt, 2016; Woodcock et al., 2011) and affect the direction and magnitude of the restoration trajectory.

The efficiency of hay transfer depends on the ability of the degraded ecosystem to enable a lasting installation of the transferred species, starting with seed germination and seedling recruitment (regeneration niche sensu Grubb, 1977). Restoration operations may be required to improve and maintain site conditions during a period of sufficient length to offer a window of opportunity for seedling recruitment (Balke, Herman, & Bouma, 2014). In floodplain ecosystems, high water availability and nutrient levels lead to strong interspecies competition (Donath, Bissels, Hölzel, & Otte, 2007; Pywell et al., 2011), which may hamper these initial recruitment steps. By decreasing competition from vegetation already standing on the degraded ecosystem or from seeds contained in the seed bank, soil preparation (e.g., topsoil removal, plowing, or harrowing) has been shown to improve the recruitment of transferred species (Jaunatre, 2012; Klanderud, Meineri, Töpper, Michel, & Vandvik, 2017; Klimkowska et al., 2010; Schmiede, Otte, & Donath, 2012; Török, Vida, et al., 2011). Mowing or grazing can also control aboveground biomass and litter accumulation and may therefore be relevant tools for grassland restoration (Billeter et al., 2008; Coiffait‐Gombault et al., 2011; Dostálek & Frantík, 2008; Pykälä, 2003; Török, Vida, et al., 2011). However, canopy opening may also favor the germination of undesirable species from the seed bank (Török, Vida, et al., 2011), while grazing can induce severe damage to seedlings through defoliation or trampling (Milchunas, Sala, & Lauenroth, 1988). Although mowing or grazing is essential to support grassland vegetation dynamics during restoration, the question thus arises as to how they can influence restoration trajectories, particularly through their effects on the recruitment of plant communities following hay transfer.

The aim of the present study was to determine the effects of early‐stage management through grazing and mowing on the restoration trajectory of previously arable land after a hay transfer operation. For that purpose, a randomized block design experiment was set up on an alluvial floodplain that had been used for intensive maize cropping for several decades. Three hypotheses were tested using this experimental design: (H1) the initiation of a restoration trajectory is strongly dependent on seed availability (dispersal) and the regeneration niche, thus the addition of seeds from the reference community (through hay transfer) associated with the control of standing vegetation and the creation of free space for germination (through harrowing) should open a window of opportunity and initiate the restoration trajectory toward the reference community; (H2) the early stages of colonization are extremely vulnerable to disturbance (trampling, grazing), thus a period without any disturbance immediately after hay transfer is necessary to prevent damage to young seedlings and keep the window of opportunity open; the recruitment of hay‐transferred species in plots protected from grazing should then be improved; and (H3) the type and timing of disturbance is likely to select differently species and therefore influence species turnover, thus the type of management (grazing or mowing) and the timing of subsequent treatments (late–early) should affect both the magnitude and the direction of the restoration trajectory.

In restoration ecology, the quality of the prediction of a trajectory will depend on both the number and the relevance of the measures on which this trajectory is based (Laughlin et al., 2017). At the community level, these measures can be both quantitative (e.g., species richness and eveness) and qualitative (e.g., specific composition) and inform about different ecosystem properties (Brudvig, 2017). Therefore, for the sake of precision, several community‐related metrics were used during this study.

## 2 MATERIALS AND METHODS

### 2.1 Study site

The study site was Raymond Island, part of a 100‐ha fluvial island located 60 km upstream of the Gironde estuary in southwestern France (44°40′38.4″N, 0°22′02.0″W). It is bordered by the main channel of the Garonne river on its west side and separated from the bank on the east by a narrow and little active Garonne annex. The island results from the connection of several small islands caused by river channeling works started in 1830. Its altitude ranges from 2 to 10 m a.s.l., and the soil displays some heterogeneity due to the aggregation of alluvium over time (silty and sand fluviosol). From the early 1900s, the island was used for fishing and agricultural activities, with a mosaic of crops, orchards, grasslands, and forests (Thébault, 2012). In the 1970s, the land was converted to intensive maize production. These farming activities lasted for almost 30 years until local government authorities acquired the 44 ha of Raymond Island in 2010 for an ecological restoration project. Because of its agricultural history and local context, this project included the maintenance of farming activities in the form of extensive sheep grazing. This required the conversion of part of the island into grassland, which led to an ecological restoration operation which included rehabilitation of all this grassland and initiation of the experiment (Corcket, Benot, Bischoff, Poncin, & Henriot, 2015).

After the last maize harvest in 2009/2010, about 30.5 ha were left as fallow in mid‐September 2010; all vegetation was destroyed by grinding with three disk crossings. A commercial organic‐labeled mixture of grassland plant species from ABE Pinault (Brittany, France) was then sown in October 2010 to rehabilitate the land. The mixture contained three Fabaceae species (*Lotus corniculatus* L., *Trifolium repens* L., and *Trifolium hybridum* L.) and three Poaceae species (*Lolium perenne* L., *Schedonorus arundinaceus* Schreb., and *Dactylis glomerata* L.). Since spring 2011, the grassland has been managed by the grazing of Scottish Blackface sheep and mechanical mowing. The flock comprises around 150–200 sheep which graze on average from March to October, although the grazing period may depend on meteorological and flooding conditions. Depending on the year and vegetation height, mechanical mowing may be performed in early summer. The grassland surface is divided into eight enclosures (four in the south of the island, numbered P1–P4, and four in the north, numbered P5–P8), enabling rotation of the flock during the grazing season.

### 2.2 Experimental design

A randomized block experiment was set up in July 2014, with one block corresponding to one enclosure. Within each enclosure (*n* = 8), we established five plots of ca. 100 m^2^, 10 m distant from each other and determined linearly following the topography so that plots within a particular enclosure were positioned at a similar altitude (*n* = 40 plots; Figure S1). Because even minor topographical variations may have substantial consequences on flooding and soil moisture patterns, the topographical position of each experimental plot was referenced by recording the altitude of each of the four plot corners using Trimble Geo 7X and then averaged. Within each enclosure, each plot was randomly assigned to one of the five management treatments under test: four plots were inoculated with hay and managed differently, that is., initial grazing (IG), delayed grazing (DG), mowing (M) and late mowing (LM), and a control without hay transfer (C).

The hay donor site was floodplain grassland managed by cattle grazing and mechanical mowing, located 15 km downstream from Raymond Island (44°45′41.6″N, 0°31′41.0″W). It is part of a “bocage” composed of species‐rich alluvial grasslands traditionally managed by cattle grazing and mowing, where protected species such as *Fritillaria meleagris* L. or *Oenanthe silaifolia* M.Bieb (Caze, Blanchard, Olicard 2006) are also found. The composition of plant species in the donor grassland was determined from four 16 m^2^ plots studied in June 2013.

In order to increase the chances of transferred seed germination and seedling establishment, the standing vegetation was mown and the soil harrowed prior to hay transfer on all the experimental plots except the controls (C). The equipment consisted in 16 vertical rotary discs that each stripped the first 5 cm of soil over a width of 75 cm. Hay was collected from the donor site on August 13, 2014, when most of the species were producing seeds and then transferred immediately to the experimental plots on the study site. The area of the hay donor grassland was approximately 9,500 m^2^. Hay transfer was based on a ratio of about 3 to 1 on 32 plots of 100 m^2^. On August 13, 2014, and August 14, 2014, about 1 m^3^ of freshly mown hay was spread by hand on each experimental plot (initial grazing, delayed grazing, mowing, and late mowing) except for control plots (C) (see image in Figure S2).

The experimental plots differed in terms of the management method applied from the time of hay transfer. The control and initial grazing plots were not fenced so they received the same management as the entire enclosure. In particular, sheep present in the enclosure could move freely through the initial grazing plots immediately after hay transfer. The delayed grazing plots were only fenced during the first year of experiment, after which they were opened to sheep grazing in June 2015 and the fences were finally removed at the end of 2015. The mowing and late mowing plots were fenced permanently to prevent any grazing. These plots were mowed yearly from 2015, in early summer (June) or late summer (late‐August to early September), respectively (see Figure S3 for mowing dates). Depending on the year and flooding conditions, annual management of the grassland may consist of sheep grazing from April to October or mowing in June followed by sheep grazing from late July–early August to October.

### 2.3 Monitoring of the vegetation

In order to obtain an accurate, objective, and easily repeatable survey of changes to the vegetation, monitoring was performed using the pin‐point method (Stampfli, 1991, 1992). The position of the data collection frame was marked using two permanent metallic pins indicating the diagonal of a 1 × 1 m^2^ quadrat. Monitoring started at this initial position and then the 1 × 1 m^2^ quadrat was moved successively three times to enable the monitoring of the whole 1 × 4 m^2^ area (Figure S4), which corresponded to the minimum survey surface for grassland.

The 1 × 4 m^2^ monitoring area was positioned at the center of each experimental plot in order to avoid any side effects (Figure S4). Vegetation monitoring was performed twice, once in May–June 2015 and once in May 2017 (Figure S3). The pin points were positioned every 25 cm, resulting in 64 points within the quadrat (Figure S4). A coefficient of 1 was attributed to the species contacted by a metal rod inserted vertically at each point, and a coefficient of 0.5 was attributed to species present within the quadrat but not touched by the rod.

### 2.4 Data analysis

In each plot, raw coefficients of the sampled species were transformed into relative abundances. The relative abundance of species *i* in quadrat *k* was calculated as follows:

$$A_{\mathrm{ik}}=N_{\mathrm{ik}}/\sum_{i=1}^{S}\left(N_{\mathrm{ik}}\right)$$

where *N_ik_* corresponds the total pin‐point coefficient of species *i* in quadrat *k* and *S* is species richness recorded within quadrat *k*. For each plot, species richness, Pielou's evenness and the community structure integrity index, CSII (Jaunatre et al., 2013) were calculated to determine the short‐term success of restoration. The CSII quantifies the average proportion of species abundance in the reference communities represented within the restored community and is defined as follows:

$$\text{CSII}=\left[\frac{\bar{\sum_{i=1\ldots S}\left(n_{i}-\Delta_{\bar{i,j}}\right)}}{\sum_{i=1\ldots S}n_{i,j}}\right]$$

With *n_i_* the abundance of species in the restored community and *n_i,j_* in the reference community, $\begin{array}{c} -\Delta_{\bar{i,j}} \end{array}$ the absolute difference between abundances in the restored and reference communities when abundance is lower in the assessed community than in the reference community, and *S* is the total number of species in the community. CSII ranges from 0 to 1: it takes a value of 1 when all species in the restored community are at least as abundant as in the reference community, and a value of 0 when there are no common species in the restored and reference communities (Jaunatre et al., 2013). CSII thus makes it possible to focus on the abundance deficit of reference species in the community under assessment.

From the initial list of species sown for grassland rehabilitation in 2010 and the plant species composition of the reference ecosystem (donor grassland) determined in 2013 (Table S1), three different species groups could be discriminated. Species recorded during the experiment in both 2015 and 2017 were classified according to these three categories (Table S2). The *Reference* species group (*RSp*) included all species encountered in the donor grassland, except for those sown on the study site during the grassland rehabilitation in 2010. These six latter species constituted the *Initial* species group (*ISp*). The remaining species that did not belong to either of these groups constituted the *Other* species group (*OSp*). The percentage of species belonging to each group was calculated for each plot based on their presence or absence.

To assess the effects of management methods and years of monitoring on species richness, Pielou's evenness, CSII index, and the percentages of each species group, linear mixed effect models (LMM) were used, with the management treatment, year and their interactions as fixed factors and the enclosure and topography as random factors. We used a log link with a Poisson error distribution for species richness and an identity link with a normal error distribution for the other variables (Crawley, 2013).

Nonmetric multidimensional scaling (NMDS) was carried out on the whole (2015 and 2017) [quadrat × species] abundance matrix in order to detect the principal differences between management treatments according to their temporal trajectories. The total frequency of each species was calculated regardless of the management treatment. Species whose total frequency of occurrence was below 5% were removed from the database, whether they were targeted in the community or not.

For each management treatment, the three most dominant species (i.e., those with the highest average abundance) and the three species with the highest indicator values were selected (Table S3). Indicator values were calculated using the *indval* function in the *labdsv* package (Roberts, 2016). The indicator value (IndVal) is a quantitative index that enables identification of the species most characteristic of a group (in this case, a management treatment) based on its fidelity (i.e., the species is present in most plots of this group) and specificity (i.e., the species is found mostly in this group rather than in other groups) (Dufrêne & Legendre, 1997).

All analyses were performed using R statistical software (version 3.4.3 R Development Core Team, 2017). Linear mixed effect models were run using the *lmer* function in the *lmerTest* package (Kuznetsova, Brockhoff, & Christensen, 2016). When necessary, multiple comparisons were run using the *CLD* function from the *emmeans* package (Lenth, Singmann, Love, Buerkner, & Herve, 2019). Nonmetric multidimensional scaling was performed using the *metaMDS* function from the vegan package (Oksanen et al., 2018), and the results were plotted using the *s.class* function from ade4 (Chessel, Dufour, & Thioulouse, 2004).

## 3 RESULTS

The average species richness per plot ranged from 8.63 ± 1.06 to 21.37 ± 7.56 in 2015 depending on the management treatment and increased significantly in 2017 (*p*‐value < .05), with values ranging from 11.13 ± 4.97 to 21.25 ± 4.59 (Table 1). Regardless of the experimental management treatment and year, the species richness of the hay‐inoculated plots was significantly higher than in control plots (*p*‐value < .01) (Table 1, see Table S3 for cumulative species richness). Pielou's evenness was significantly affected by interactions between the year and management treatment (*p*‐value < .05). The lowest evenness was recorded for the control management treatment in 2017, and the highest values were reached under the initial grazing and late mowing management treatments in 2017 (Table 1).

**Table 1:** Mean ± *SE* species richness and evenness under each experimental treatment in 2015 and 2017

	Treatments				
	C	IG	DG	LM	M
Species richness					
2015	8.63 ± 1.06	21.37 ± 7.56	18.37 ± 3.66	15.87 ± 3.23	15.75 ± 6.25
2017	11.13 ± 4.97	21.25 ± 4.59	20.25 ± 4.98	18.37 ± 4.44	18.75 ± 4.23
Evenness					
2015	0.66^bc^ ± 0.10	0.70^ab^ ± 0.05	0.72^ab^ ± 0.05	0.69^abc^ ± 0.04	0.73^ab^ ± 0.05
2017	0.61^c^ ± 0.05	0.75^a^ ± 0.07	0.73^ab^ ± 0.04	0.75^a^ ± 0.03	0.72^ab^ ± 0.03

Note

Lower case letters indicate significant differences.

Abbreviations: C, control; DG, delayed grazing; IG, initial grazing; LM, late mowing; M, mowing.

Both dominant and indicator species reflected temporal changes to the vegetation and management effects (Table S4). In 2015, the vegetation under all management treatments was dominated by *S. arundinaceus*, *D. glomerata,* or *L. perenne*, corresponding to the *initial species group*, and *P. trivialis*, which belonged to the *other species group* (Tables S4 and S5). In 2015, no indicator species was recorded under delayed grazing, late mowing, and mowing treatments, and only one species, which belonged to the *initial species group* (*S. arundinaceus*), was an indicator of the control treatment, while initial grazing was the only treatment characterized by three grassland species belonging to the *reference species group* (*Oenanthe pimpinelloides*, *Holcus lanatus,* and *Hordeum secalinum*). In 2017, the three strongest indicator species under the initial grazing treatment still came from the *reference species group*, while *S. arundinaceus* remained one of the indicator species of the control treatment. At that date, indicator species under the mowing and late mowing treatments also emerged: while the late mowing treatment was characterized by species belonging to the *reference species group*, the mowing treatment was characterized by ruderal species from the *other species group*. No indicator species was detected with the delayed grazing management treatment. Finally, in 2017, while *S. arundinaceus* remained dominant regardless of the treatment, some *reference species* also became dominant in all treatments, except the control.

**Figure 1:**
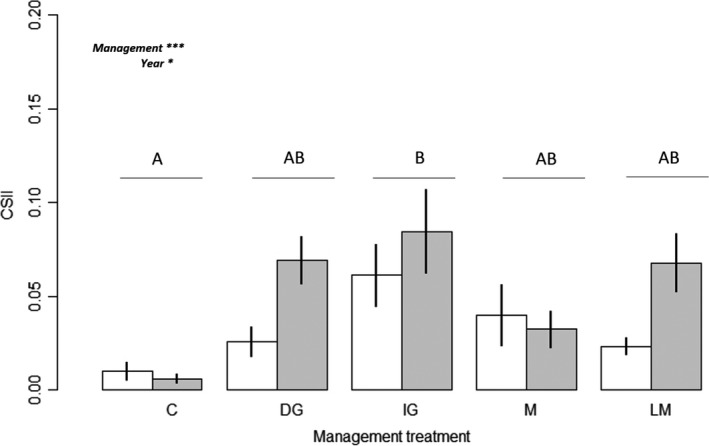
Mean (±*SE*) community structure integrity index (CSII) values in 2015 (white) and 2017 (gray) according to the five experimental treatments. C, control; DG, delayed grazing; IG, initial grazing; LM, late mowing; M, mowing. Different letters indicate significant differences between treatments

The community structure integrity index (CSII) was significantly higher in 2017 than in 2015 (*p*‐value < .05) regardless of the management treatment applied (Figure 1). Whatever the year, the CSII was significantly higher under the initial grazing treatment than with the control treatment. However, the maximum average CSII value after 3 years of monitoring was still low (0.09 ± 0.06 with the initial grazing management treatment).

**Figure 2:**
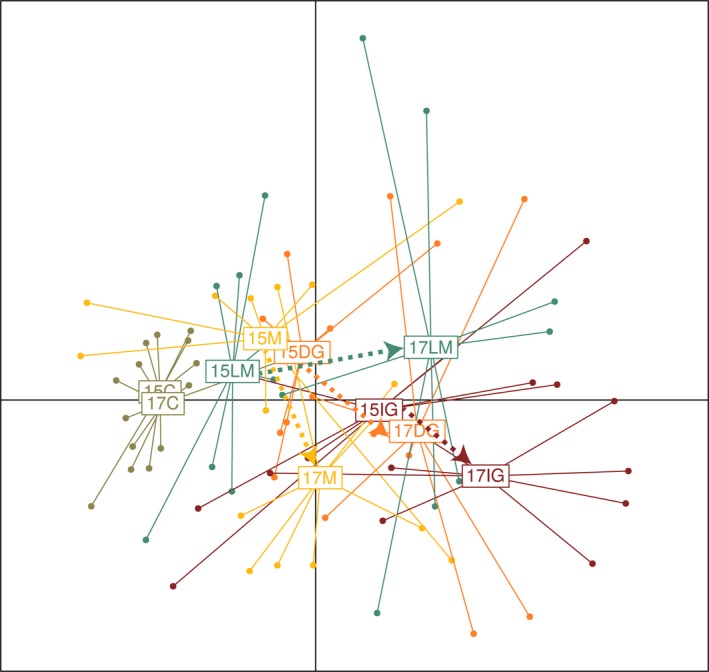
Nonmetric multidimensional scaling (NMDS) plot based on the Bray–Curtis dissimilarity of species composition between each experimental plot (40 plots × 5 treatments × 67 species); NMDS axis 1 is horizontal and NMDS axis 2 is vertical. Vegetation records are grouped according to year and management treatment. C, control; DG, delayed grazing; IG, initial grazing; LM, late mowing; M, mowing; 15: year 2015, 17: year 2017. Arrows represent vegetation dynamics between 2015 and 2017

Projection of the plots on the NMDS axis 1–NMDS axis 2 factorial plane revealed a distinction between the years and management treatments, despite heterogeneity within each management treatment and year (Figure 2). Nonmetric multidimensional scaling axis 1 mostly reflected a year effect, with a shift between 2015 and 2017 directed toward the positive side of this axis, while NMDS axis 2 mainly seemed to discriminate between the management treatments (Figure 2). However, these patterns, and particularly the magnitude and direction of temporal change, were dependent on the management treatment. First, almost no temporal change was observed with the C treatment, which remained on the negative side of NMDS axis 1. Plots within this control treatment appeared to be quite homogeneous. The temporal changes affecting the mowing, delayed grazing, and initial grazing treatments occurred along both the NMDS axis 1 and the NMDS axis 2, being even more marked along this second axis for mowing, while the shift tended to occur along the NMDS axis 1 for the late mowing treatment. In 2015, both axes enabled discrimination between the management treatments. The control and initial grazing treatments were separated from each other and from the three other management treatments, which were less clearly discriminated. Initial grazing in 2015 was positioned at the level of the NMDS axis 1, similar to the other hay‐inoculated treatments in 2017. In 2017, all treatments were more clearly discriminated from each other, along both NMDS axis 1 and NMDS axis 2.

**Figure 3:**
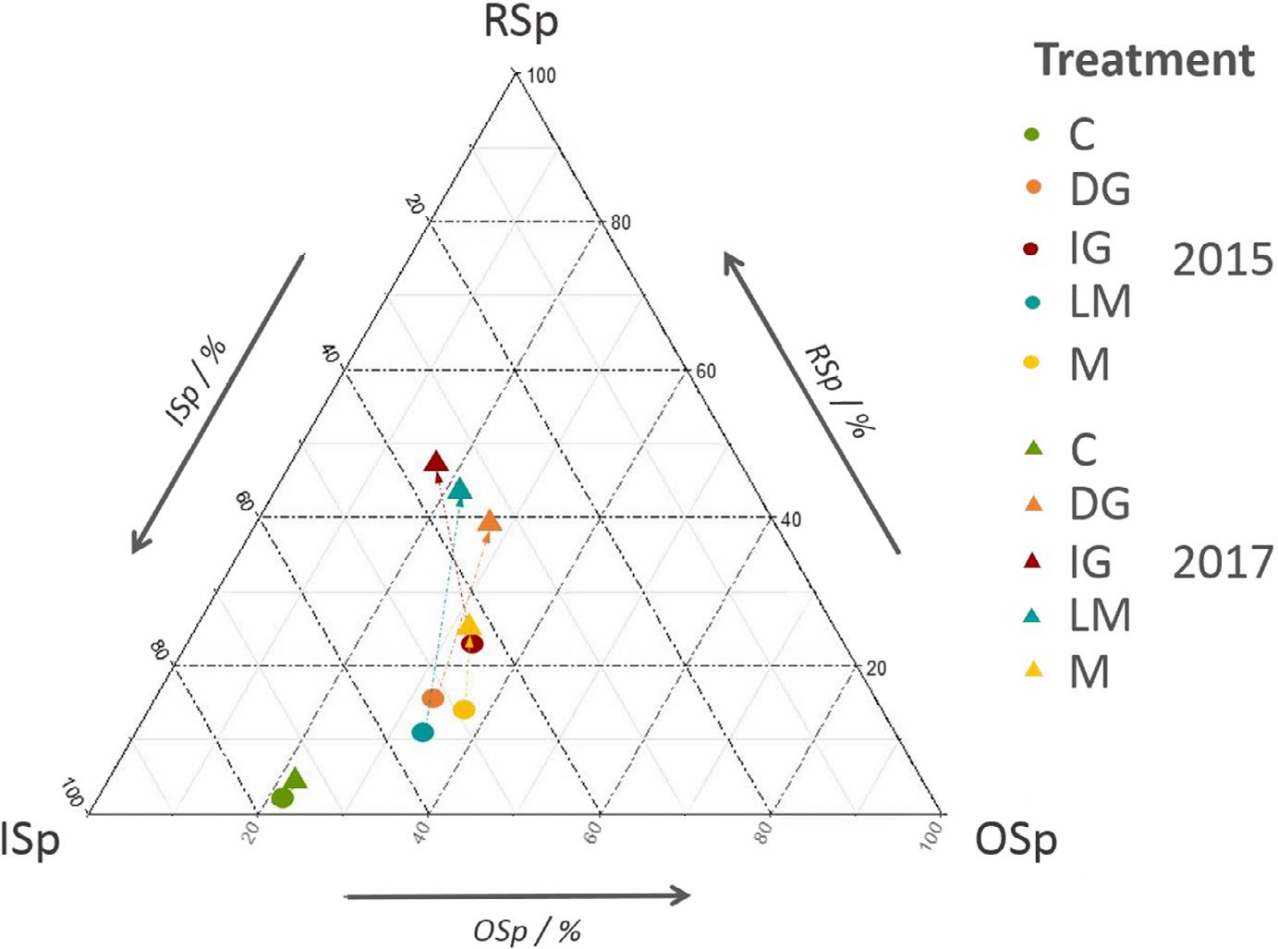
Ternary plot of species groups represented in each treatment during the two monitoring years, 2015 (circles) and 2017 (triangles). Arrows represents the dynamic of each treatment between 2015 and 2017. C, control; DG, delayed grazing; IG, initial grazing; ISp, Initial species group; LM, late mowing; M, mowing; OSp, Other species group; RSp, Reference species group

The ternary plot enabled formalization of the temporal trajectory of the five treatments in terms of species proportions. In both 2015 and 2017, vegetation under the control treatment was mainly composed of the *initial species group* but also contained 21.3% ± 10.8% of species from the *other species group* (Figure 3, Table S6). Compared with the control treatment, hay inoculation significantly increased the proportion of the *reference species group*, with the initial grazing treatment only in 2015 but also with all other hay‐inoculated treatments in 2017 (Figure 3, Table S6). For all treatments except the control, the temporal dynamics from 2015 to 2017 were oriented toward the *reference species group*, but to different degrees (Figure 3). This was reflected by a significant reduction in the proportion of the *initial species group* under delayed grazing (from 51.7 ± 13.3% to 33.5 ± 10.4%) and late mowing (from 54.9 ± 5.9% to 34.3 ± 11.0%), and a significant increase in the proportion of the *reference species group* under initial grazing (from 23.8 ± 17.2% to 47.7 ± 17.5%), delayed grazing (from 15.9 ± 5.5% to 38.8 ± 12.4%), and late mowing (from 11.4 ± 7.3% to 44.5 ± 12.2%), whereas no significant temporal change to the proportions of species was recorded for mowing plots (Table S6).

## 4 DISCUSSION

### 4.1 Effects of harrowing and hay transfer: opening a window of opportunity for seedling recruitment

Ten months after hay transfer and regardless of the treatment, the vegetation was still dominated by the grasses sown during the initial rehabilitation stage 4 years previously, that is, *S. arundinaceus* and *D. glomerata*. These results confirm the strong influence of the vegetation matrix constituted during rehabilitation, which could exert a competitive effect and act as an efficient filter against plant recolonization. However, a few other species, such as *Poa trivialis*, *Helminthotheca echioides,* or *Sonchus asper*, which originated from either the seed bank or seed rain, also managed to establish themselves, probably favored by regeneration niches opened up by sheep grazing during the 4 years after the rehabilitation stage (Török, Vida, et al., 2011).

In accordance with hypothesis H1, our results showed that hay transfer was able to initiate changes in the vegetation, as reflected by significantly higher species richness and evenness, along with a generally lower proportion of species sown during the rehabilitation stage (*initial species group*) in all hay‐inoculated plots when compared to control plots. Such anticipated positive effects of hay transfer have been widely reported in wet grasslands (Klimkowska et al., 2007; Moreno‐Mateos, Meli, Vara‐Rodríguez, & Aronson, 2015; Sengl et al., 2017), at mine sites (Baasch, Kirmer, & Tischew, 2012), mediterranean (Coiffait‐Gombault et al., 2011; Jaunatre, 2012), and calcareous grasslands (Kiehl, Thormann, & Pfadenhauer, 2006). During our experiment, this hay transfer effect was linked to an increase in the proportion of both *reference* (*RSp*) and *other* (*OSp*) species, most of which are characteristic of fallow plant communities. Thus, the gain in species richness observed as from the first year of the experiment did not only result from hay transfer but also from expression of the seed bank that had probably been prevented previously by the strongly competitive vegetation cover generated by rehabilitation. This suggests that soil harrowing prior to hay transfer generated suitable site conditions for seedling recruitment (regeneration niche sensu Grubb, 1977) and opened a window of opportunity that benefited both inoculated and soil seed bank species (Hofmann & Isselstein, 2004; Török, Vida, et al., 2011).

### 4.2 First‐year management effects on the window of opportunity

The grassland under study has been managed by sheep grazing since its rehabilitation in 2010. Because defoliation and trampling linked to grazing can cause direct damage to plants (Belsky, 1987; Lagendijk, Howison, Esselink, Ubels, & Smit, 2017; Milchunas et al., 1988), enclosure of the hay‐inoculated plots, at least during the first months after transfer (delayed grazing, late mowing and mowing plots), was expected to favor seedling recruitment by protecting young seedlings from such negative grazing effects (hypothesis H2). But contrary to this expectation, initial grazing was the only hay‐inoculated treatment that resulted in a significantly higher proportion of *reference species* and CSII than the control treatment. The initial grazing treatment was also characterized by three indicator species from the *reference species* group, as from the first year of the experiment. Thus, as early as 2015, this management treatment appeared to be characterized by plant species assemblages similar to those attained under other hay‐inoculated treatments in 2017 (as reflected by their positions along NMDS axes). By contrast, in 2015, fenced plots (delayed grazing, late mowing, and mowing) were intermediate between the control and initial grazing plots in terms of several vegetation structure and composition metrics (e.g., position on the NMDS axis, proportions of different species groups, CSII, and indicator species). These results suggest that rather than negative direct effects on seedlings, sheep grazing immediately after hay transfer exerted a positive effect on the germination and installation of seedlings. These unexpected results were probably linked to the timing of grazing, which had been applied on the experimental plots for a few months immediately after hay transfer (August–October 2014), that is, a period during which the germination of seeds contained in the hay had not yet been initiated. Not only did trampling probably favor seed germination (Winkel & Roundy, 1991), but also defoliation did not directly impact the transferred species at that development stage. On the contrary, it is likely that defoliation controlled the regrowth of standing vegetation, especially from vegetative buds that had not been destroyed by harrowing, although it could stimulate plant growth in the short term (Corcket & Moulinier, 2012). Under such conditions, the window of opportunity was kept open by the grazing‐induced control of aboveground competition rather than by a disturbance‐free period, as might have been expected (e.g., Balke et al., 2014).

### 4.3 Three‐year management effects on the restoration trajectory

We expected the type and timing of management (grazing or mowing) applied to influence the magnitude and direction of the restoration trajectory (hypothesis H3). Because the delayed grazing, late mowing, and mowing treatments were all applied as from spring 2015, their effects could only be reflected by the 2017 survey. Accordingly, the discrimination of plant species assemblages among these three management treatments could only be assessed in 2017. While the vegetation of all hay‐inoculated treatments displayed temporal dynamics, species assemblages in the control plots tended to be similar in 2015 and 2017, and the proportion of each species group did not change significantly between 2015 and 2017. Indeed, grassland dynamics following the cessation of cropping and even rehabilitation toward reference ecosystems are known to last for at least a few decades if no additional restoration operations are implemented (Török, Kelemen, et al., 2011; Török, Vida, et al., 2011). Long‐term monitoring of our plots is continuing in order to confirm the trajectory imprinted by the control plots without any hay transfer.

Unlike the control treatment, the temporal dynamics of the community composition of hay‐inoculated plots were of greater magnitude and resulted in 2017 in the dominance of at least one *reference* species (*RSp*) under all hay‐inoculated treatments and a significantly higher proportion of *reference* species (*RSp*) in 2017 than in 2015, except for the mowing treatment. As for the indicator species in 2017, three of them were in the *other species group* characteristic of postcultural fallow plant communities (*Convolvulus arvensis*, *H. echioides,* and *S. asper*) under the mowing treatment. By contrast, two *reference* species were indicators of late mowing and three *reference* species were indicators of the initial grazing treatment. These results suggest that the establishment of *reference* species was not only affected by the window of opportunity opened during the very early stages of the restoration operation but also by the type of management applied subsequently. While sheep grazing and mowing are expected to reduce aboveground competition and create regeneration niches (Grubb, 1977; Klimešová, Janeček, Bartušková, Lanta, & Doležal, 2010; Török et al., 2016) for both established species and those from the seed bank, these effects may vary depending on several factors, such as their intensity or timing. For instance, sheep grazing likely maintains regeneration niches throughout the growing season, leading to a reduction in competition between species, while mowing has a short‐term effect by consistently removing biomass (Hofmann & Isselstein, 2004; John, Dullau, Baasch, & Tischew, 2016; Tälle et al., 2016). The only difference between the mowing and late mowing treatments lies within their timing (June and September, respectively): Because it is applied earlier in the growing season, mowing is likely to damage early‐growing species and favor those with late growth, while late mowing will mainly remove the biomass of late‐growing species, thereby creating regeneration niches for the germination of seeds dispersed earlier in the season. However, even if a *reference* species is present in the community, its relative abundance does not necessarily reflect the reference grassland, as suggested by very low community structure integrity index values.

### 4.4 Conclusion and perspectives: recommendations for restoration operations

Soil harrowing and the regeneration filters induced by the management treatments applied during our in situ experiment exerted significant effects on seedling recruitment. We nevertheless found evidence of the effects of management methods applied during the early stages of grassland restoration by hay transfer regarding the initiation of plant community dynamics toward reference ecosystems. In particular, although the early temporal dynamics of plant communities after hay transfer were roughly oriented toward reference ecosystems, we observed a divergence of floristic composition between the four hay‐inoculated treatments. If a restoration operation is designed to accelerate the establishment of *reference* species, one can strongly recommend management techniques that will keep windows of opportunity open between seed transfer and germination. During the present study, this was achieved by sheep grazing, which probably also had positive effects on seeds due to trampling (initial grazing treatment). This method should, however, be applied with caution and match the timing between hay transfer and seedling recruitment, in order not to damage young seedlings. Traditional grassland management based on defoliation by grazing or mowing may further support community dynamics toward reference ecosystems.

Practitioner managing restoration operations should therefore consider three essential features of these trajectories: origin, magnitude, and direction. In our experiment, the origin of a trajectory was determined not just by the hay transfer operation but also by the management applied at the same time. Subsequent management treatments seemed to influence both the magnitude and the direction of the trajectory. Future investigations are therefore necessary and would, in particular, benefit from functional approaches. Monitoring of our study plots is continuing in order to determine the importance of these initial stages of restoration to long‐term trajectories.

## CONFLICT OF INTEREST

None declared.

## AUTHOR CONTRIBUTIONS

E.C., M.‐L.B., and D.A. conceived the idea and designed the experiment. E.C., M.‐L.B., and D.A. took part in installing the experiment. All authors collected field data. M.G., M‐L.B, and D.A analyzed the data and wrote the manuscript. All the authors contributed to the draft and gave final approval to the manuscript.

## Data Availability

We agreed to deposit our data in a public repository: it will be available on Dryad from the following DOI accession number (https://doi.org/10.5061/dryad.crjdfn30r).
