# Supplementary material for: The effects of management on vegetation trajectories during the early‐stage restoration of previously arable land after hay transfer
Source: Ecol Evol. 2019 Dec 6;9(24):13776–86. doi: 10.1002/ece3.5798 (PMC6953687; doi:10.1002/ece3.5798)
Supplement: Supplementary file 1 [file ECE3-9-13776-s001.docx]

# Supporting information


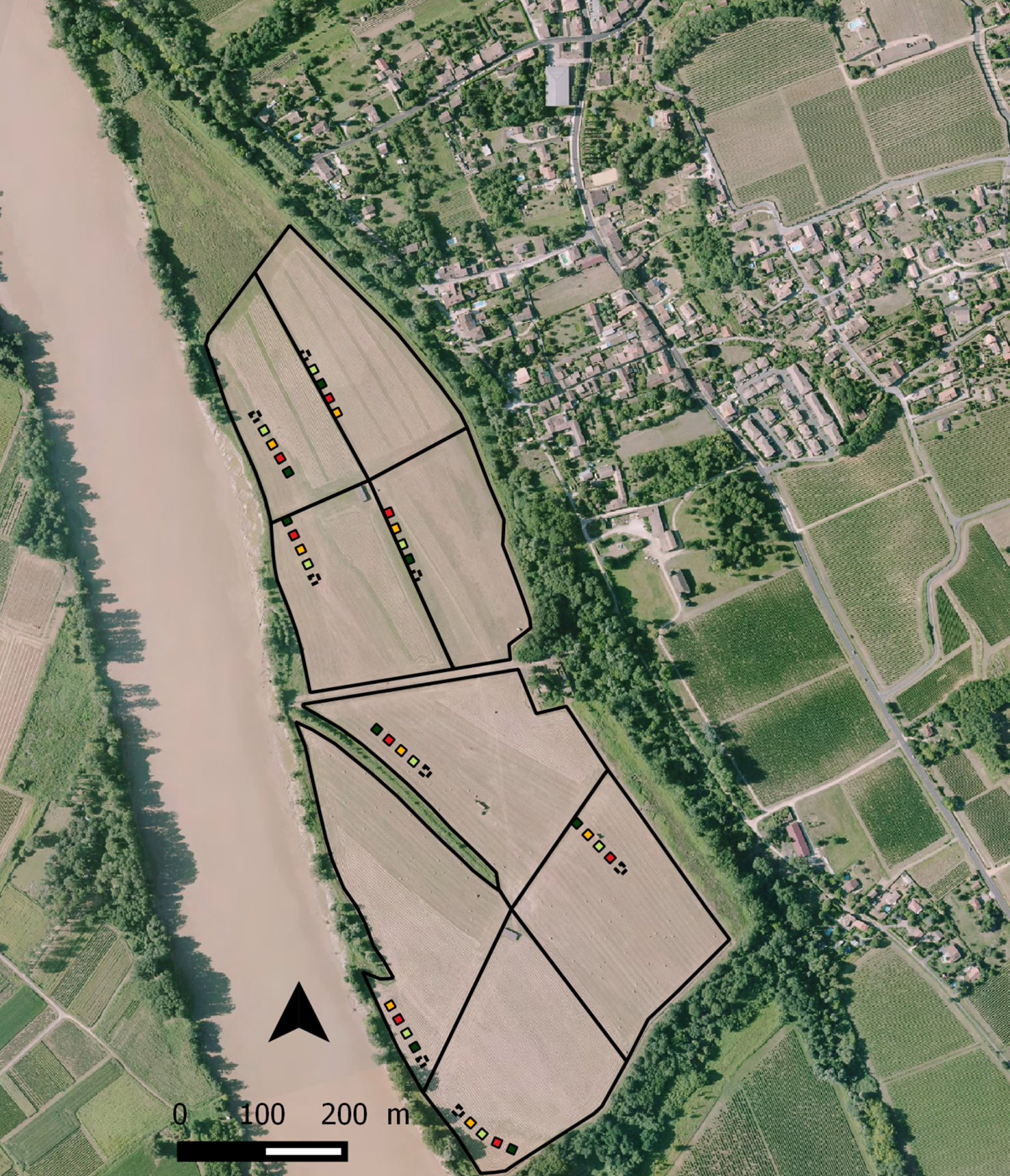


P3

P5

P8

P7

P6

P4

P1

P2


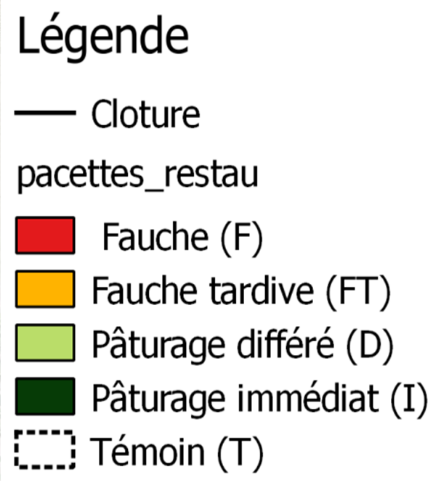


Mowing (M)

Late Mowing (LM)

Delayed Grazing (DG)

Initial Grazing (IG)

Control (C)

Fences

Legend

**Figure S1** Map of the Ile de Raymond grassland representing the eight enclosure, the location of the 40 experimental plots and the corresponding management treatments.


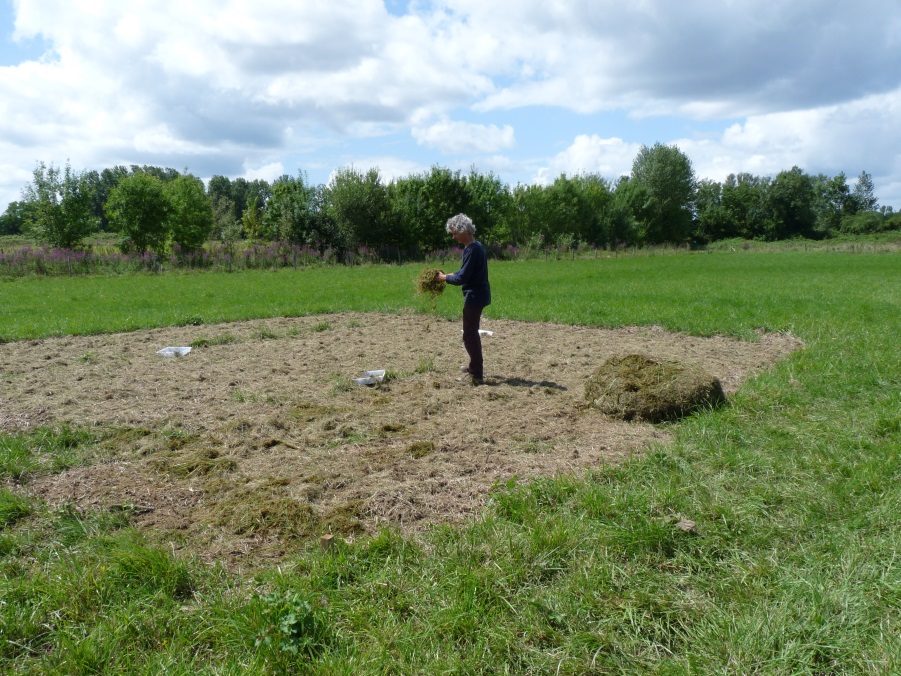


**Figure S2** Hay scattering in the experimental plots after the transfer from the reference grassland


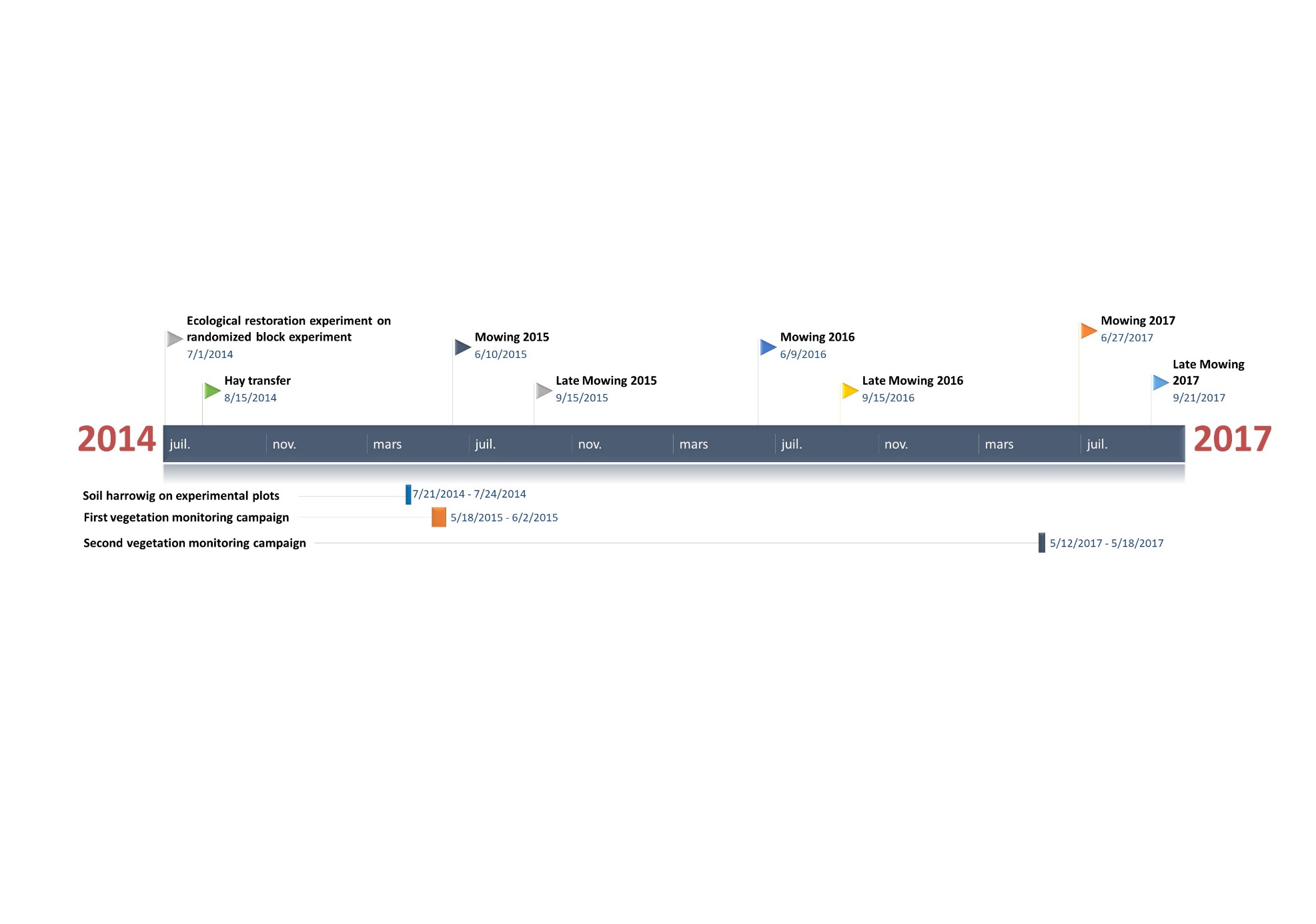


**Figure S3** Calendar of hay transfer and experimental events.


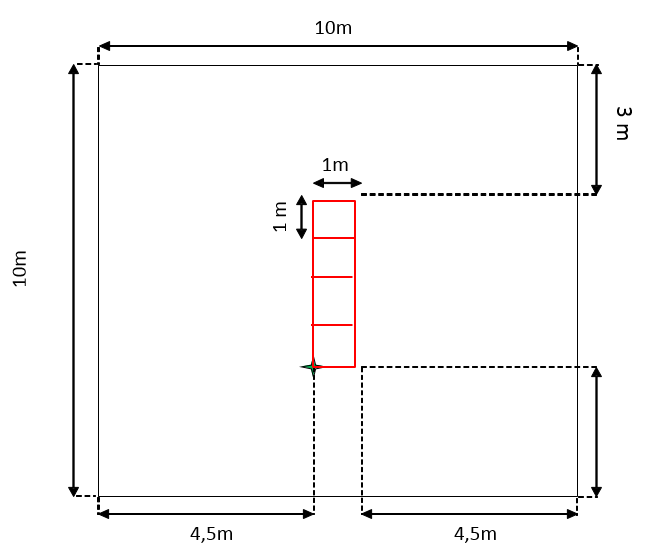


South

North

**Figure S4** Sampling methods for vegetation monitoring: location of the four 1 x 1 m pin-point quadrats (in red), and of the two metallic pins (green crosses) materializing the first quadrat position.

**Table S1**. Reference grassland species list.

| ***Species*** |
| --- |
| *Agrostis sp* |
| *Alopecurus pratensis* |
| *Althaea officinalis* |
| *Anthoxanthum odoratum* |
| *Arrhenatherum elatius* |
| *cf Crepis sp* |
| *Bromus racemosus* |
| *Calystegia sepium* |
| *Carex cf riparia* |
| *Carex divisa* |
| *Carex flacca* |
| *Carex hirta* |
| *Carex cuprina* |
| *Carex tomentosa* |
| *Centaurea thuillieri* |
| *Cerastium fontanum* |
| *cf Fraxinus excelsior* |
| *cf Lycopus europaeus* |
| *Cynosurus cristatus* |
| *Cyperus longus* |
| *Dactylis glomerata* |
| *Elytrigia repens* |
| *Epilobium tetragonum* |
| *Festuca cf ovina* |
| *Galium debile* |
| *Galium verum* |
| *Gaudinia fragilis* |
| *Geranium dissectum* |
| *Holcus lanatus* |
| *Hordeum secalinum* |
| *Juncus acutiflorus* |
| *Juncus conglomeratus* |
| *Lathyrus nissolia* |
| *Lathyrus pratensis* |
| *Leucanthemum vulgare* |
| *Linum bienne* |
| *Lolium perenne* |
| *Lotus corniculatus* |
| *Lotus tenuis* |
| *Lotus uliginosus* |
| *Lythrum salicaria* |
| *Medicago cf arabica* |
| *Mentha suaveolens* |
| *Myosotis laxa* |
| *Oenanthe pimpinelloides* |
| *Orchis laxifolia* |
| *Pastinaca sativa* |
| *Plantago lanceolata* |
| *Poa trivialis* |
| *Potentilla reptans* |
| *Prunella vulgaris* |
| *Ranunculus acris* |
| *Ranunculus flammula* |
| *Ranunculus repens* |
| *Rhinanthus angustifolius* |
| *Rumex acetosa* |
| *Rumex conglomeratus* |
| *Rumex crispus* |
| *Festuca arundinacea* |
| *Silene flos-cuculi* |
| *Stellaria alsine* |
| *cf Trisetum flavescens* |
| *Trifolium patens* |
| *Trifolium pratense* |
| *Trifolium repens* |
| *Vicia sativa* |

**Table S2**. List of species encountered in experimental design at both years 2015 and 2017.

| ***Restoration site species*** | ***Group*** |
| --- | --- |
| *Agrostis sp* | Reference |
| *Anthoxanthum odoratum* | Reference |
| *Apiaceae sp* | Other |
| *Arrhenatherum elatius* | Reference |
| *Asteraceae sp* | Other |
| *Bromus sp* | Reference |
| *Centaurea thuillieri* | Other |
| *Cerastium glomeratum* | Other |
| *Cirsium sp* | Other |
| *Convolvulus arvensis* | Other |
| *Cynosurus_cristatus* | Reference |
| *Dactylis glomerata* | Initial |
| *Dipsacus fullonum* | Other |
| *Elytrigia repens* | Reference |
| *Epilobium sp* | Other |
| *Equisetum arvense* | Other |
| *Erig sp* | Other |
| *Ervum tetraspermum* | Other |
| *Fraxinus excelsior* | Other |
| *Galium aparine* | Other |
| *Galium sp* | Reference |
| *Gaudinia fragilis* | Reference |
| *Geranium dissectum* | Reference |
| *Hedera helix* | Other |
| *Helminthotheca echioides* | Other |
| *Holcus lanatus* | Reference |
| *Hordeum secalinum* | Reference |
| *Hypochaeris radicata* | Other |
| *Lapsana communis* | Other |
| *Lathyrus nissolia* | Reference |
| *Lathyrus pratensis* | Reference |
| *Linum usitatissimum subsp. angustifolium* | Reference |
| *Lolium perenne* | Initial |
| *HYBRIDE* | Other |
| *Lotus corniculatus* | Initial |
| *Lotus glaber* | Reference |
| *Medicago cf arabica* | Other |
| *Medicago lupulina* | Other |
| *Medicago sativa* | Other |
| *Myosotis sp* | Other |
| *Oenanthe pimpinelloides* | Reference |
| *Ophrys apifera* | Other |
| *Plantago lanceolata* | Reference |
| *Poa trivialis* | Other |
| *Polygonum sp* | Other |
| *Potentilla reptans* | Reference |
| *Prunella vulgaris* | Reference |
| *Ranunculus acris* | Reference |
| *Ranunculus repens* | Reference |
| *Raphanus raphanistrum subsp landra* | Other |
| *Rubus sp* | Reference |
| *Rumex acetosa* | Reference |
| *Rumex crispus* | Reference |
| *Schedonorus arundinaceus* | Initial |
| *Sonchus sp* | Other |
| *Taraxacum officinale* | Other |
| *Trifolium dubium* | Other |
| *Trifolium hybridum* | Initial |
| *Trifolium patens* | Reference |
| *Trifolium pratense* | Reference |
| *Trifolium repens* | Initial |
| *Valerianella_locusta* | Other |
| *Veronica officinalis* | Other |
| *Veronicasp* | Other |
| *Vicia sativa* | Other |

**Table S3**. Cumulative number of species in each treatment (at both dates 2015 and 2017) and the reference grassland.

|  | **Treatment** | | | | | **Reference** |
| --- | --- | --- | --- | --- | --- | --- |
|  | C | IG | DG | LM | M | Cadaujac |
| **cumulative number of species** | 34 | 59 | 58 | 52 | 53 | 66 |

**Table S4**. Raunkiaer types (G geophytes, Hc hemicryptophytes, Th Therophytes), growing period (main period, which can extend from / to months indicated into brackets) and main habitats (from Tison and de Foucault 2014) for species identified as either dominant or indicator species for each management treatment and year (see Table S2). The family and group (Reference species group, Initial species group, Other species group) are also indicated.

| **Species** | **Family** | **Group** | **Raunkiaer type^*^** | **Growing period^**^** | **Main habitats** |
| --- | --- | --- | --- | --- | --- |
| ***Agrostis capillaris*** | Poaceae | Reference | Hc | Jun – Sep | Grasslands, heathlands, understorey vegetations |
| ***Agrostis stolonifera*** | Poaceae | Reference | Hc | Jun – Sep | Grasslands, fallows |
| ***Bromus cf. commutatus*** | Poaceae | Reference | Th | May – Jun | Grasslands, fallows |
| ***Centaurea decipiens*** | Asteraceae | Reference | Hc | Jun – Oct | Grasslands, heathlands, herbaceous forest fringes |
| ***Holcus lanatus*** | Poaceae | Reference | Hc | May – Jul – (Oct) | Grasslands |
| ***Hordeum secalinum*** | Poaceae | Reference | Hc | May – Jul | Grasslands |
| ***Lathyrus nissolia*** | Fabaceae | Reference | Th | Mar – Jun | Grasslands, rarely fallows |
| ***Oenanthe pimpinelloides*** | Apiaceae | Reference | Hc / G | May – Jul | Grasslands, understorey vegetations |
| ***Dactylis glomerata*** | Poaceae | Initial | Hc | (Jan) – Mar – Jul – (Dec) | Grasslands, herbaceous forest fringes |
| ***Lolium perenne*** | Poaceae | Initial | Hc | Apr – Oct | Grasslands |
| ***Lotus corniculatus*** | Fabaceae | Initial | Hc | Apr – Aug – (Oct) | Grasslands and other open to semi-closed habitats |
| ***Schedonorus arundinaceus*** | Poaceae | Initial | Hc | Apr – Jul – (Nov) | Grasslands, herbaceous forest fringes |
| ***Convolvulus arvenis*** | Convolvulaceae | Other | G | May – Oct | Gardens, grasslands, fallows |
| ***Epilobium hirsutum*** | Onagraceae | Other | Hc | Jun – Sep | Crops, fallows |
| ***Epilobium tetragonum*** | Onagraceae | Other | Hc | Jun – Oct | Tall herb fringes, grasslands |
| ***Helminthotheca echioides*** | Asteraceae | Other | Th / Hc | May – Nov | Fallows |
| ***Ophris apifera*** | Orchidaceae | Other | G | May – Jul | Grasslands |
| ***Poa trivialis*** | Poaceae | Other | Hc | Apr – Jul – (Oct) | Grasslands, fallows, herbaceous forest fringes, understorey vegetations |
| ***Sonchus asper*** | Asteraceae | Other | Th | Mar – Sept | Fallows |

**Table S5.** Dominant and indicator species for each experimental treatments in 2015 and 2017. The five experimental treatments are « M », mowing, « LM », late mowing, « DG », differed grazing, « IG », initial grazing and « C », control.


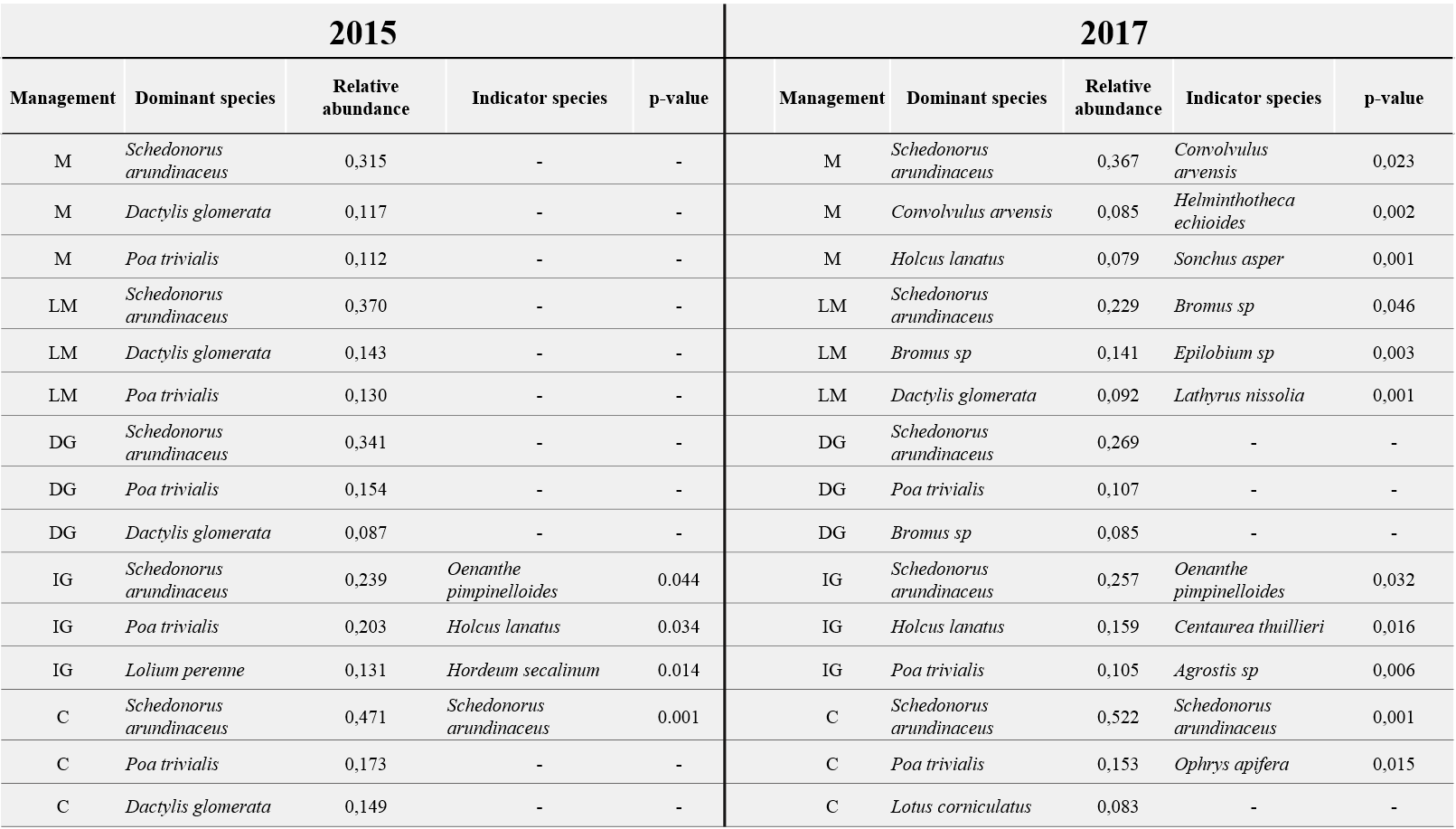


**Table S6.** Species group represented on monitoring samples in percentage for each treatment in 2015 and 2017. “C”: control, “DG”: delayed grazing, “IG”: initial grazing, “M”: mowing, “LM”: late mowing. The letters show significant differences.

|  | | **Treatment** | | | | |
| --- | --- | --- | --- | --- | --- | --- |
|  |  | **C** | **IG** | **DG** | **M** | **LM** |
| **Reference species (%)** | *2015* | 1.0^a^ ± 1.0 | 23.8^b^ ± 17.2 | 15.9^ab^ ± 5.5 | 14.0^ab^ ± 10.7 | 11.4^ab^ ± 7.3 |
|  | *2017* | 2.3^a^ ± 3.8 | 47.7^d^ ±17.5 | 38.8^cd^ ± 12.4 | 25.0^bc^ ± 9.1 | 44.5^d^ ± 12.2 |
| **Initial species (%)** | *2015* | 77.7^a^ ± 11.0 | 43.3^bcd^ ± 26.1 | 51.7^bc^ ± 13.3 | 49.5^bcd^ ± 13.6 | 54.9^ab^ ± 5.9 |
|  | *2017* | 75.1^a^ ± 14.4 | 35.4^bcd^ ± 17.8 | 33.5^d^ ±10.4 | 43.3^bcd^ ± 6.8 | 34.3^cd^ ± 11.0 |
| **Other species (%)** | *2015* | 21.3^abc^ ± 10.8 | 32.9^a^ ± 10.7 | 32.4^abc^ ±11.9 | 36.4^abc^ ± 10.3 | 33.7^ab^ ± 12.0 |
|  | *2017* | 22.5^abc^ ± 11.9 | 16.9^bc^ ± 4.7 | 27.7^bc^ ± 10.4 | 31.8^bc^ ± 8.7 | 21.2^c^ ± 15.6 |
